# Health Behaviors of Austrian Secondary Level Pupils at a Glance: First Results of the *From Science 2 School* Study Focusing on Sports Linked to Mixed, Vegetarian, and Vegan Diets

**DOI:** 10.3390/ijerph182312782

**Published:** 2021-12-03

**Authors:** Katharina C. Wirnitzer, Clemens Drenowatz, Armando Cocca, Derrick R. Tanous, Mohamad Motevalli, Gerold Wirnitzer, Manuel Schätzer, Gerhard Ruedl, Werner Kirschner

**Affiliations:** 1 Department of Subject Didactics and Educational Research and Development, University College of Teacher Education Tyrol, 6010 Innsbruck, Austria; derrick.tanous@student.uibk.ac.at (D.R.T.); mohamad_motevali@yahoo.com (M.M.); 2 Department of Sport Science, University of Innsbruck, 6020 Innsbruck, Austria; armando.cocca@uibk.ac.at (A.C.); Gerhard.Ruedl@uibk.ac.at (G.R.); Werner.Kirschner@uibk.ac.at (W.K.); 3 Research Center Medical Humanities, Leopold-Franzens University of Innsbruck, 6020 Innsbruck, Austria; 4 Division of Sport, Physical Activity and Health, University of Teacher Education Upper Austria, 4020 Linz, Austria; clemens.drenowatz@ph-ooe.at; 5 adventureV & change2V, 6135 Stans, Austria; gerold@wirnitzer.at; 6 Special Institute for Preventive Cardiology and Nutrition—SIPCAN, 5061 Salzburg, Austria; m.schaetzer@sipcan.at

**Keywords:** pupil, student, physical activity, plant-based, health, fruit, vegetables, nutrition, lifestyle

## Abstract

Attaining healthy behaviors is essential at any life stage, particularly childhood, due to the strong link between children’s lifestyle and the subsequent adult state of health. This multidisciplinary study aimed to assess lifestyle behaviors of Austrian pupils of secondary schools I and II, with a specific focus on PA habits and diet types based on a large sample. In total, 8845 children/adolescents participated in the short standardized online survey on relevant health-related aspects nationwide. Valid and complete data was provided by 8799 pupils, including 1.14% of the eligible 771,525; 63% girls, 76% having a normal body weight, 70% attending secondary schools II, and more pupils/students living in rural vs. urban areas (3:1 ratio). Across the total sample, 11.8% were considered overweight/obese with a higher prevalence of overweight/obesity in boys than girls (15.5% vs. 9.6%) and urban vs. rural participants (13.9% vs. 10.8%; *p* < 0.05). The majority of participants (84.5%) reported a mixed diet, while 7.2% and 8.5% reported a vegan and vegetarian diet, respectively. Vegans reported a lesser alcohol intake (*p* < 0.05) compared to non-vegan pupils (no difference in dietary subgroups for smoking). Although overall PA and dietary behaviors suggest an appropriate health status among Austrian youth, attention should be focused on policies to increase healthy lifestyle habits at best through a dual approach to health permanently combining regular PA, sports, and exercise with a healthy diet, which would contribute to matching the current recommendations for improving individual and public health.

## 1. Introduction

Health is an essential component of any individual’s life [1], which assumes higher importance at early ages [2]. Indeed, there exists a strong chance of showing positive health status through adulthood if a person experienced a healthy lifestyle during childhood [3]. On the contrary, children experiencing early health issues may drag them throughout their life and develop more serious diseases during adulthood [4]. Hence, a primary objective for researchers and practitioners from different fields is to monitor youth’s health status and the factors that may positively or negatively affect it. Several lifestyle factors have been identified as potential mediators of health in childhood. Physical activity (PA) and healthy dietary patterns are considered cornerstones towards building a positive health status [5,6].

The effect of PA on health in youth is well documented. For instance, PA in young people results in improved cardiovascular function [7], cognitive performance, and attentional capacity [8]. Further, PA conducted either indoors or outdoors has been associated with enhanced general psychosocial health [9]. Similar positive effects have been reported in studies on youth with ongoing health issues, such as attention-deficit/hyperactivity disorder (ADHD) or mental health problems [10,11].

An important aspect when considering PA in youth is its organization, i.e., structured (through sports clubs, physical education, etc.) or unstructured PA (leisure time activities). Structured PA has been shown as highly effective for youth health, as it may avoid the risks associated with the unstructured form when this is carried out without adult supervision and with no clear skill-building focus [12]. Likewise, Krustrup et al. suggest that participation in organized sports, in and out of school, may lead to an improved health profile in children [13]. Participation in PA, sports, and exercise is essential for developing appropriate physical and mental health in youth [14]. As mentioned above, together with organized sports, unstructured leisure time activities constitute an essential factor for improving or deteriorating children’s health, depending on the nature of such activities. When including active behaviors, such unstructured leisure time activities have been shown to positively affect one’s state of health [15]. High sedentary time, such as excessive screen time, on the other hand, is significantly associated with long-term illness [15]. Ekblom-Bak et al. [16], for example, emphasized the importance of leisure time PA in association with physical education and organized sports as a pillar of long-term health [17].

As has been addressed for PA, proper nutrition is also known to be essential for both children’s growth and health status [18]. In the last decades, special attention has been given to the type of diet adopted by individuals and the different effects on health. In particular, researchers are investigating the differences, health effects, advantages, and detrimental health outcomes of omnivorous, vegetarian, and vegan diets. According to the Academy of Nutrition and Dietetics (AND), well-planned vegetarian and vegan diets that include all necessary nutrients for proper growth are healthful, nutritionally adequate, and appropriate at all life stages [19]. Such diets can also provide significant preventive and therapeutic effects against different chronic diseases, such as type 2 diabetes or certain types of cancers [20,21], with additional benefits of plant-based diets towards community health [22]. Children and adolescents adhering to these diets have been shown to obtain an adequate nutrient intake, whereas omnivorous youth tend to consume fewer vegetables in favor of dairy and fats [23]. Independent of diet type, the assurance of an adequate intake of all essential nutrients has been emphasized to avoid the risk of undersupply and/or deficiencies in the growth process [24]. In this sense, daily intakes of fruit and vegetables [25], as well as sufficient fluid intake and hydration [26], are considered key elements of any diet type, often studied together with alcohol consumption and tobacco use [27].

Consequently, the analysis of PA and diet, especially their continuous interaction, together with social-environmental and individual factors influencing such interaction, e.g., Body Mass Index (BMI) [28], living environment [29], or sex [30] becomes a vital step for a better understanding of the health status of any young population. However, the scientific data available on the current dietary trends (e.g., plant-based diets) is lacking, particularly (1) on children and adolescents and (2) on the school context. This multidisciplinary study aimed to examine lifestyle behaviors of Austrian students at secondary levels I and II, with a specific focus on “PA/sport” and “diet” across different subgroups.

## 2. Materials and Methods

### 2.1. Study Protocol and Ethics Approval

*From Science 2 School* (www.science2.school/en, accessed on 26 November 2021) was designed as a cross-sectional study (multidisciplinary approach using a multi-level cluster sampling strategy) and was conducted Austria nation-wide with a large sample. This Austria nation-wide study is supported by the Federal Ministry of Education, Science, and Research, Department 1/7—School and University Sports. The study protocol was approved by the ethics board of all nine Austrian Federal Education Authorities (Bildungsdirektionen), which was mandatory to contact the principals of 2688 secondary schools (levels I and II) across Austria as a final step of approval to conduct the study in the classrooms. Due to the requests of the respective Austrian federal education authorities and the respective school management, no further ethical vote (e.g., the institutional review board or local ethical committee) was required for this study. The interested reader is kindly referred to the study protocol [31].

### 2.2. Participants

**Figure 1:**
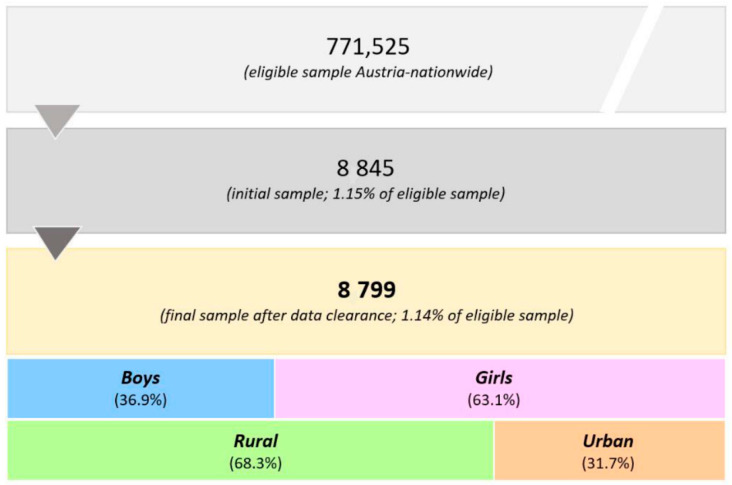
Flow chart of the pupils’ enrollment and classifications based on sex (boys vs. girls) and living area (rural vs. urban).

The target group was all pupils of secondary levels I and II, resulting in a basic sample size of 771,525 pupils. Accordingly, all secondary schools (levels I and/or II: n = 2688) were contacted via E-mail to participate in the study. The school management received the initial information regarding the goal and procedure of the study and transferred this information to the class directors for surveying pupils throughout regular classroom sessions. At the closure of data collection (10 July 2020), a total number of 8845 children and adolescents participated in the online survey. Figure 1 shows the participants’ enrollment.

### 2.3. Procedures

Pupils of Austrian secondary school levels I and II were asked to complete a standardized online questionnaire via an encrypted interface (available/provided in German). Before the study, participants received written information about the study procedure and gave written informed consent to participate in the study. Participation was anonymous and voluntary, which could be withdrawn by the participant at any time without the provision of reasons or negative consequences. Under the supervision of their teachers, school principals, or parents, the pupils filled in the questionnaire comfortably at school or home, via smartphone, tablet, or PC/laptop.

This self-report survey consisted of five parts with questions about the individual, including age and anthropometric characteristics (part A), PA & sports (part B), nutrition (part C), health (part D), and miscellaneous (part E). Several control questions were included in different parts of the questionnaire to identify conflicting information and increase the reliability of data sets.

**Figure 2:**
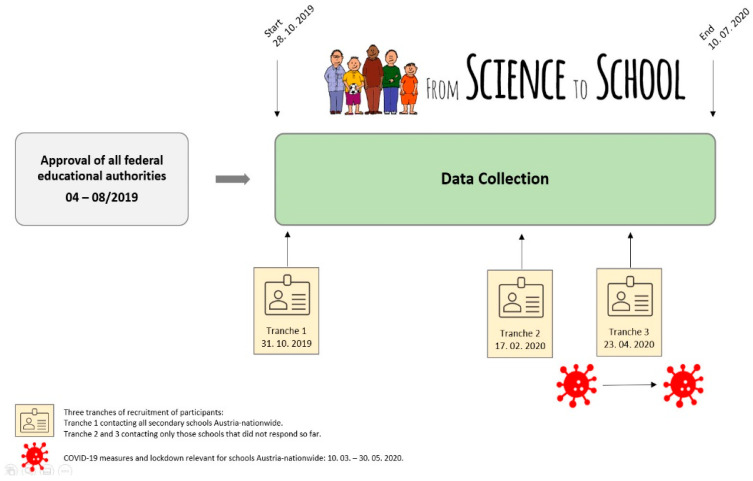
Flow chart of procedure and timescale of a previous application to approval of educational authorities and subsequent data collection by online survey.

Regarding the operational implementation, two steps were required: (1) approval of the questionnaire for implementing the survey directly at the schools themselves in all nine Austrian federal states by the respective state school boards of the nine Austrian federal educational authorities; and (2) support by the Federal Ministry of Education, Science, and Research to facilitate contact with the participating schools. Figure 2 shows the procedure (recruitment of participants was accomplished by three tranches) and timescale of a previous application of the approval by educational authorities and the subsequent data collection via the online survey.

### 2.4. Measures

The survey was conducted Austria nation-wide and collected data on socio-demography (nationality, age, sex, federal state, residence (urban vs. rural); and region); anthropometry (height, body weight, calculated BMI (kg/m^2^)); secondary school level I or II, school type; nutrition (e.g., fruit and vegetable, fluid, current diet type), including smoking and alcohol consumption and PA. Based on the diet report, participants were classified as vegetarian (devoid of meat and processed meat inclusive fish and shellfish, but intake of dairy, eggs, honey), vegan diet (devoid of all foods and ingredients from animal source), or mixed/omnivore (no dietary restrictions). For PA, participants reported their engagement in sports and exercise (e.g., duration/day, frequency/week, type of sport, organizational form, competition participation, member of a sports club), and leisure time activities.

Participants with a bodyweight of less than 20 kg, height less than 110 cm, or those with calculated BMI values of <10 kg/m^2^ or >50 kg/m^2^ (deemed implausible) were removed from data analysis. BMI was converted to BMI percentiles (BMI_PCT_) using German reference values with the 90th and 97th percentile serving as cut-points for overweight and obesity, respectively; participants with a BMI_PCT_ below the 10th percentile were classified as underweight [32].

### 2.5. Data Clearance

From the total number of 8845 participants, 46 pupils were excluded from the study due to incompatible reports on height (n = 26), body weight (n = 9), and calculated BMI (n = 11). After data clearance, a total of 8799 children and adolescents (1.14 % of the eligible 771,525) were included in the final data analysis. Statistically reliable, representative results (based on data regarding demographics/biometrics, e.g., sex, age, school level, PA levels, and diet type were calculated to be detectable with a minimum sample size of n = 984. Further detailed information is available in the study protocol [31].

### 2.6. Statistical Analysis

Descriptive statistics were calculated, and data are reported as mean with standard deviation for continuous data and prevalence for nominal data. Differences in anthropometric characteristics and age by living environment (urban vs. rural), sex, school type, sports participation, and nutrition were examined via multivariate analysis of variance (MANOVA). Differences in sports participation and dietary pattern by living environment and migration background were examined via chi-square tests. Further, chi-square tests were used to examine differences in dietary pattern (e.g., intake of fruits, vegetables, and fluids), smoking and alcohol use by sports participation, and diet type (mixed vs. vegetarian vs. vegan diet). Chi-square tests were also used to examine differences in sports participation by diet type. All statistical tests were performed with SPSS 26.0 (SPSS Inc., IBM Corp., Armonk, NY, USA). The level of statistical significance was set at *p* ≤ 0.05.

## 3. Results

The final sample consisted of 8799 participants (36.9% male, 63.1% female) from all nine federal states of Austria. The distribution of participants by the living situation, nationality, and school attended is shown in Table 1. More than 2/3 of the participants were in secondary school level II (starting with grade 9), while the rest of the participants attended secondary level I schools (grades 5 through 8). Across the entire sample, 68.3% lived in rural areas, and 88.0% were Austrian. The most common nationalities among non-Austrian participants came from Turkey (17.7%), Germany (11.4%), Serbia (10.2%), Bosnia and Herzegovina (9.4%), Hungary (9.3%), Croatia (6.7%), and Romania (6.0%).

### 3.1. Anthropometric Characteristics

Anthropometric characteristics across the total sample and by sex and school type (secondary level I vs. secondary level II) are shown in Table 2. Supplementary results provided in Table A1 and Table A2 further show anthropometric data by federal state and school level, separately for urban and rural areas. Despite being younger than female participants (*p* < 0.01), male participants were significantly taller and heavier than their female peers (*p* < 0.01). Male participants also had a higher BMI_PCT_, which was also reflected by a higher prevalence of overweight and obesity (*p* < 0.01), and the prevalence of underweight was higher for female participants (*p* < 0.01). Further, BMI_PCT_ had a linearly decreasing trend across age groups (*p* < 0.01) from an average of 50.6 ± 30.7 between the ages 10 and 12 years to 44.1 ± 31.2 between the ages 17 and 19 years. Similarly, the prevalence of overweight/obesity declined with increasing age (*p* < 0.01) from 12.2% between the ages 10 and 12 years to 10.1% between the ages 17 and 19 years. Accordingly, participants in secondary level I displayed a higher BMI_PCT_ than participants in secondary level II. Participants living in urban and rural areas also differed in their anthropometric characteristics, which resulted in a higher prevalence of overweight and obesity in urban areas (*p* < 0.01).

### 3.2. Sports Participation

The distribution of physical activity engagement is displayed in Table 3, while Table 4 displays anthropometric characteristics by sports participation. A majority of participants (82.4%) reported regular sports participation during their leisure time, but less than half the participants (42.5%) were active members of sports clubs. Across the entire sample, participants engaged in sports on 2.9 ± 2.0 days a week with a higher sports participation in male participants compared to females (*p* < 0.01). More male participants also reported regular leisure-time sports participation and participating in club sports (*p* < 0.01). Participation in recreational sports and club sports decreased with increasing age (*p* < 0.01), which also resulted in a decline in the number of days participants reported playing sports (*p* < 0.01). Accordingly, leisure time and club sports participation rates differed significantly by school level (*p* < 0.01), with a prevalence of 90.2% vs. 79.1% and 48.9% vs. 39.3% for leisure time and club sports, respectively. Austrian participants also reported higher sports participation compared to participants of another nationality (*p* < 0.01), but there was no difference in sports participation between participants living in rural and urban areas. Participants reporting regular engagement in sports were younger than their peers, which also resulted in lower body weight and height (*p* < 0.01). BMI_PCT_, however, did not differ by sports participation. Nevertheless, sports participation was associated with a lower prevalence of overweight and obesity (*p* < 0.01).

### 3.3. Diet

Dietary pattern distribution is displayed in Table 5, with Table 6 showing anthropometric characteristics by dietary pattern. Based on the reported dietary intake, most participants (84.3%) consumed a mixed diet, with a comparable number of participants consuming a vegetarian or vegan diet. A mixed diet was more common in male participants compared to females. However, a vegan diet was more common in males than a vegetarian diet, while the opposite was observed in female participants. Despite being the dominant dietary pattern in urban and rural areas, a mixed diet was more common in rural areas. On the other hand, vegan diets were more common in participants living in urban environments as compared to their peers living in rural environments. While mixed diets were more common in secondary school level II pupils compared to participants from secondary school level I (87.0% vs. 78.2%), vegan diets were more common in pupils of secondary school level I compared to secondary school level II (14.3% vs. 4.1%). Accordingly, participants reporting a vegan diet were significantly younger than participants reporting a mixed or vegetarian diet (*p* < 0.01). Furthermore, Austrian participants more commonly reported a mixed diet compared to participants of another nationality, while non-Austrian participants had a greater rate of adhering to a vegan diet.

Participants with a vegetarian diet displayed the lowest BMI_PCT_ (*p* < 0.01), but there was no significant difference in the prevalence of overweight across dietary patterns. The adiposity rate, however, was significantly lower in participants reporting a vegetarian diet (*p* < 0.01), and underweight was more common in participants with a vegetarian or vegan diet compared to participants with a mixed diet (*p* < 0.01).

### 3.4. Physical Activity and Diet

Table 7 displays the association between sports participation and diet-related health behaviors. Sports participation, particularly during leisure time, was associated with a higher prevalence of daily fruit and vegetable consumption and fluid intake of more than 2 L/day (*p* < 0.01). While generally the most common choice, water was more often reported as the primary fluid consumed in participants with regular leisure-time sports. Soft drinks and diluted juices, on the other hand, were more common in participants reporting no regular recreational sports. After considering age differences in sports participation, smoking was less common in pupils reporting recreational sports participation, but there was no difference between club sports and non-club sports participants. Alcohol consumption also did not differ by club sports participation or engagement in recreational sports.

Regular leisure-time sports participation was more common in participants reporting a vegan diet (*p* < 0.01), but there was no difference in club sports participation across dietary patterns (Table 8). Accordingly, participants with a vegan diet reported a higher number of days per week with sports participation in comparison to their peers (vegan diet: 3.2 ± 2.1 days/week; vegetarian diet: 2.9 ± 2.0 days/week; mixed diet 2.8 ± 2.0 days/week).

While there was no difference in the total fluid intake across dietary patterns, participants with a vegetarian reported water more often as the primary drink compared to their peers. Diluting juices and soft drinks, on the other hand, were less often reported as the primary fluid choice in participants with a vegetarian diet. No difference was observed for smoking behavior across dietary patterns, but participants with a vegan diet reported lower alcohol consumption (Table 8). Additional information on sports participation, eating behaviors, alcohol consumption, and smoking prevalence by federal states and living environment is provided in Table A3 and Table A4.

## 4. Discussion

This study aimed to assess PA habits linked to current diet types of Austrian children and adolescents in secondary school levels I and II based on a large sample. This school study is the first to examine the dual approach to health behavior with a specific focus on the most important lifestyle factors, “PA, sports and exercise” and “diet,” interwoven and associated with sex, BMI, living area, as well as alcohol and smoking habits, and is based on representative data from a total of 8799 children and adolescents.

More females than males participated in the survey (2 in 3 or 63%), along with approximately 70% of participants attending secondary school II and more pupils living in rural vs. urban areas (ratio of 3:1). Approximately 76 % (3 in 4) of the total sample were found having a normal body weight considered as healthy [32,33,34], with 84 % following a mixed diet, and a higher prevalence of those involved in leisure and club sports (77% and 80%, respectively) vs. non-actives. The most important findings are: (a) ~12% prevalence of overweight/obesity in significantly more boys (higher BMI_PCT_) than girls (ratio of 3:2) and living in urban compared to rural areas; (b) 82.4% of pupils reported to engage in leisure time sports and 42.5% were engaged in club sports with an average of ~3 days per week, while sports participation was found to be more popular in boys than girls without any significant difference between rural and urban areas but declining with age; (c) ~16 % prevalence of vegetarians (8.5%; 7.2% vegans) with more than twice the number of vegetarian girls than boys when compared to vegan and mixed diets, which were more popular among boys; (d) vegan and vegetarian pupils were found to be significantly more active in leisure-sports than omnivores (about 86% and 84% vs. 82%), and vegetarians had a significantly lower BMI_PCT_ compared to vegan and omnivorous pupils; (e) daily intake of healthy dietary items (fruit, vegetables, water) was found significantly higher in club and leisure sports vs. non-actives, especially when the weekly PA frequency increases; (f) a significant reduction in alcohol intake with the increase of weekly PA frequency; and (g) significantly less alcohol consumption in vegan vs. non-vegan pupils but no significant difference between dietary subgroups considering smoking habits.

Western and developing civilizations worldwide are facing two large-scale health problems of urgent concern to be addressed not only for adults but all the more for the young, namely physical inactivity or insufficient PA and overweight/obesity [1,35,36,37,38,39,40,41,42]. However, it is well-accepted that poor lifestyle behaviors and habits track over time from the young age into adulthood and have contributed to the appearance of non-communicable diseases (inclusive of their risk factors) in adults but nowadays occurring at young ages, too [2]. For instance, about 30% of Austrian children and adolescents were reported having excess body weight [43,44,45,46,47]. In this context, and with the world facing the current COVID-19 pandemic (ongoing since 03/2020), which already affects pupils and students markedly and seems to result in a detrimental impact on shaping healthy behaviors of children/adolescents now and for future generations, health is a key topic in education—and consequently human development—, in line with the UN Sustainable Development Goals (SDGs; “Good Health and Well-Being” and “Quality Education”) and UNESCO “Cross-cutting key competencies” as a central educational objective to help educators and policymakers aim at and integrate these into educational settings (e.g., curricula) [48,49,50], as well as meet the WHO Voluntary Global Targets on NCDs (especially “10% reduction in insufficient PA“) [51,52,53].

However, the present findings show that boys had higher BMI_PCT_ compared to their female peers, representing a higher percentage of overweight and obese boys than girls. These results are in line with comparable studies reporting that the percentage of overweight boys tends to be nearly three times higher than girls [54]. A cross-sectional study by Carayanni et al. [55] with a sample of 5144 children aged 12 to 15 years old found that boys are 2.9 times more likely to be overweight or obese compared to girls, which confirms the present findings. However, inconsistent results are shown in similar studies by Lisowski et al. [56], who reported no sex differences in the prevalence of overweight in children, and by Kantanista et al. [57], indicating BMI may not be a significant factor for boys or girls when planning health-focused PA programs in adolescents aged 14 to 16 years old. Moreover, Nevill et al. [58] suggested that BMI may not properly reflect changes in body fat percentage. Indeed, these authors confirm that there might be differences in BMI by sex even after adjustment of percent body fat. In Austria, it has been estimated that 30% of children/adolescents suffer from overweight/obesity [43,47] and are at risk of serious chronic health conditions (e.g., hypertension, high cholesterol levels, type 2 diabetes, respiratory disease, cardiovascular disease) passing from childhood to adulthood [59]. Zhou et al. [60] pointed out that youth living in urban areas are more likely to be overweight or obese compared to their peers living in smaller cities and rural areas, regardless of sex, which is in line with the findings on rural/urban areas in the present study. The authors proposed that while overweight and obesity are increasing in big and medium-sized cities, a potential reason for such difference depends on nutritional habits and status. Similar outcomes are shown in studies on preschoolers [61] and adults [62]; however, McCormack et al. [63] analyzed data from Health Departments across the United States and revealed that rates of obesity and overweight were higher among rural youth. The study emphasizes the need for consistently defining “rural” and the degree of rurality, as outcomes may significantly differ based on that.

As expected, in the present study, students engaged in either leisure-time exercise and/or sports clubs showed a better weight profile compared to their inactive peers. While it has been reported that 81% of Austrian children and adolescents do not reach the minimum recommended PA levels (60 min per day), two-thirds of them do not eat sufficient nutrient-rich foods (e.g., fruit and vegetables), which are proposed to contribute to the increased prevalence of overweight and obesity [43,44,45]. Hilpert et al. [64] analyzed variables influencing children’s health status in a sample of 997 participants. Their study draws attention to the fact that low sports activity levels represent a key factor in determining children’s overweight and obesity. Similarly, leisure time sedentary activities were associated with elevated body weight in German children [65]. These findings are further confirmed by Godakanda et al. [66], who underlined that the main risk factors of obesity in children were low leisure-time PA and adhering to sedentary behaviors such as screen time. In line with the present outcomes, the beneficial role of leisure-time PA is pointed out by a 6-year longitudinal study on school-aged children [67]. The authors concluded the significant impact of both physical education and leisure-time PA in reducing the detrimental consequences of developing obesity or overweight across the study period.

In the present study, pupils adhering to a vegetarian diet showed a lower incidence of obesity compared to omnivores and vegans. Although the effects of vegetarian, vegan, and omnivorous diets on physical characteristics are not yet well-established, some researchers suggest that an omnivorous diet may be significantly linked with elevated BMI compared to the vegetarian diet [68]. This finding could be linked to the fact that vegans/vegetarians were generally shown to have a higher level of health consciousness than omnivores in terms of being more active and consuming less alcohol/smoking [69,70]. Some authors have pointed out that while both vegetarian and omnivorous diets may be equally effective for losing/controlling weight, the vegetarian one seems to lead to further benefits in terms of reducing low-density lipoprotein cholesterol (LDL) levels [71,72], which may partially help to explain our results. In line with the above-mentioned study, Garousi et al. [73] reported that a vegetarian diet may be more effective than traditional diets for weight loss purposes. However, our results cannot be entirely interpreted to indicate a healthier weight profile among dietary groups, as the number of underweight vegetarians was significantly higher than underweight omnivores (but not vegans). This finding may result in some potentially dangerous repercussions, as previous research has underlined; in fact, being underweight may not only increase the risk of certain diseases in young people [74] but it has also been shown as detrimental as obesity in the development of COVID-19 related acute symptoms, illnesses and injuries [75].

Although the combined effect of a healthy diet and PA patterns on individuals’ weight status is known [76], there is no clear evidence that PA may have had a role in our results on vegetarian’s weight profile, as omnivorous, vegetarian, and vegan participants in this study showed similar habits in sports activities. Regarding healthy dietary patterns, the current Health Behavior of School-Aged Children (HBSC) reports indicate that a considerable number of children and adolescents consume insufficient nutrient-rich foods (e.g., 48% of adolescents neither eat fruits nor vegetables; daily consumption of fruits and vegetables are only 60% and 62%, respectively), which results in the failure of most children and adolescents to meet the healthy nutritional recommendations [43,44,45]. It has been estimated that 10% of Austrian adults follow vegetarian (~800,000) and vegan (~80,000) diets [77,78], and the worldwide trend of plant-based diets are growing especially in the young (12%) due to health and environmental issues [22,79], which nicely corresponds to the present results.

Concerning the linkage between movement patterns and nutritional habits, our findings suggest that pupils engaging in any kind of PA, either unstructured or organized sports, tend to have a better dietary profile, regardless of the general type of diet they adhere to. In particular, these pupils showed a higher intake of fluid, fruit, and vegetables and consumed a lower amount of alcohol. These results are in line with those reported by Mitri et al. [80], who surveyed PA and dietary habits of 798 adolescents and revealed that those engaged in high levels of PA had significantly improved diet quality. The potential role of PA in the choice of healthy nutrition is highlighted by Beaulieu et al. [81], who investigated the effects of a 12-week exercise program on overweight and obese participants and found a lower tendency to overeat or to consume high-fat food, and by Joo et al. [82] who suggest that physical exercise may increase individuals’ motivation to pursue healthier dietary preferences. However, it seems that low levels of PA may trigger behavioral traits indirectly fostering overconsumption through increased body fat [83]. Generally, it seems that there is still a gap in the literature, and more research is needed to clarify the mechanisms in which PA may influence appetite control and dietary preferences.

However, health promotion—preferably via “PA, sports, and exercise” and “healthy nutrition”—is declared as general educational objective and thus overarching scholastic principle by law from the state mandate of the Austrian curricula of secondary school levels I and II, and is relevant to each compulsory subject [84,85,86,87,88].

In order to ensure better future individual health, it is necessary to improve PA levels through joyful and motivating activities such as a wide variety of different kinds of sports (including attractive trend sports), disciplines, and exercises while promoting healthful (plant-based) diets and variations by offering, for example, healthy food items, dishes, and menus at the school canteen and buffet, as well as healthier school feeding programs. As a minimal recommendation to maintain good health and promote sustainable lifelong health, a special focus must be applied on the permanent connection of the “healthy eating—active living” dual approach into everyone’s daily schedule, since it may offer the most promising key intervention to shape good health status [79], and thus should be transferred and integrated into the pupils’ daily schedule within the school and family settings, which remains crucial in order to ensure better future public health.

Results of *From Science 2 School: Sustainably healthy—active & veggy* will add a considerable input to the current scientific literature on the topic and provide a foundation of evidence on dietary prevalence, including vegetarian and vegan diets particularly, and the permanent connection to PA, sports, and exercise within the Austrian secondary school context (levels I and II). In addition, the results will address potential health risks in a more tailored way that is dependent upon pupil dietary patterns and exercise habits. Both lifestyle behaviors are well-known to (i) contribute to the individual’s state of health for better or even for worse and (ii) serve as an intervention that is basic and low-cost but also safe and highly effective for improving pupil health [31,89]:

1. Justify the need for this dual approach to decision-makers, which should be the minimum recommendation according to the Austrian state mandate;
2. motivate policy and decision-makers in the educational context (federal authorities, school principals and teachers, families) to reassess current health-related school offerings in order to build on or even create new programs, opportunities, and materials encompassing this dual approach for everyday school scenarios (cafeteria and catering, vending machines, interdisciplinary events, etc.);
3. establish health-oriented action competence and sustainable action readiness regarding improvements to the current and long-term health status of school pupils (for pupils of all socioeconomic backgrounds).

Thus, a more direct transfer of scientific evidence to the public will be entrenched in secondary schools to expand, first and foremost, individual health with highly progressive public health benefits for nations like Austria to follow.

In this regard, it should be noted that two subsequent follow-up studies are already in plan and under design: (a) in order to map the interwoven prevalence of current dietary trends linked to levels of sports and exercise among pupils of secondary school levels I and II, we intend to transfer this study to different countries among European nations and/or EU member states that will help to provide an overview of the impact of the relationship between vegetarian, vegan, and omnivorous diets linked to sports and exercise in youth; and (b) from the original study and its conclusive statistical hypothesis, a small cohort (*From Science 2 School 2.0*) will be followed, including clinical measures (focusing on e.g., inflammation and immune defense, hair cortisol samples, changes in the vascular system, and more) with matching samples (age, sex, BW, diet type, PA levels). Further, a more experimental approach could also be implemented to (c) examine potential changes in nutritional habits at different exercise levels, as well as to test changes in other correlated health behaviors depending on the type of physical activity, weekly engagement, and intensity in groups of adolescents adhering to different diet patterns. Furthermore, the use of longitudinal designs may help us analyze how and if the trends in physical activity engagement over the years affect youth’s nutritional choices over the long term.

Moreover, from the background of the *From Science 2 School* study, the immediate follow-up study, *Sustainably healthy—From Science 2 Highschool & University* (similar cross-sectional design following the original school study: Austria nationwide), was created to overcome the lack of information and bridge the gap between the state mandate of the Austrian secondary school curricula and the specialized studies for pedagogy and teacher training at Austrian universities and colleges. *From Science 2 Highschool & University* aims to survey lecturers/academic staff and students (as future teachers, therapists, and physicians, among other specialized health, nutrition, sports, and life science professions) and addresses a special focus on the prevalence of sports and physical exercise linked to different kinds of diets within Austrian colleges and universities. In addition, both studies, *From Science 2 School* and *From Science 2 Highschool and University*, are currently underway to be carried out at the Europe/EU level.

Although the present study is the first to survey the current prevalence of various diet types linked to levels of PA, sports, and exercise among Austrian pupils at secondary school levels I and II, some limitations have to be mentioned. In addition to the fact that not all Austrian schools with all their pupils were within reach of the chosen recruitment method, the present study shares—with other studies—the following limitations: (1) the design of the cross-sectional study; (2) the likelihood of socially desired over-reporting (e.g., longer duration of PA, higher consumption of healthy food items) or under-reporting (e.g., lower body weight, lower consumption of unhealthy food items); (3) although the application of BMI percentiles method provides an accurate, age-, and a sex-adjusted indication of fat mass accumulation in children and adolescents [90], this assessment method is known to have drawbacks [91]; and (4) the sample (Austria nationwide) allows for generalization of results for Austria only but could be similar with comparable cultural and geographical regions such as Germany and Switzerland (i.e., the DACH-countries). Therefore, factors such as culture, environmental characteristics, and different school systems may lead to different lifestyle-related outcomes. In addition, the global challenge of COVID-19 (inclusive lockdowns) might have affected the public, schools, and universities in the later leg of the study with measures put into action in March 2020 (overnight, so to speak), and although of urgent concern and highly relevant to the school setting and this study, it was not possible to take into account this unpredictable situation within the online survey without any risk or consequences potentially affecting the data gathered (e.g., loss of data due to stopping and restarting the online survey, conflicting data sets of prior vs. during vs. post COVID-19 situation, getting biased data, etc.).

## 5. Conclusions

This study provides viable information on key lifestyle behaviors given the importance of PA and diet on one’s state of health, resulting in a nation’s state of health since the personal health of children and adolescents tracks over time into adulthood and old age. On average, even though Austrian children and adolescents showed good health conditions (BMI, regular engagement in PA and sports activities, healthful dietary patterns), reported PA levels were below international recommendations, and less than 2/3 of the pupils reported daily fruit and vegetable intake. In addition, almost half of the pupils reported alcohol consumption, and almost 10% smoked. However, attention should be put on the disadvantageous lifestyle behaviors considering unhealthy dietary patterns, sedentary behaviors, and a relatively considerable number of smokers and alcohol consumers in this sensitive age group.

This data emphasizes the need for continued efforts to facilitate healthy lifestyle choices in Austrian children and adolescents and can serve as a starting point for future interventions to improve health in children and adolescents. The findings may also be particularly helpful when establishing actions for improving public health status using the dual approach of healthy kinds of diet combined with healthy and regular PA, sports, and exercise.

The present findings can be used by families, teachers, trainers/coaches, health specialists, nutrition and sports experts, as well as decision-makers and multipliers in health and education. Future research on healthier and sustainable diets (particularly plant-based diets), as well as sports and exercise habits, should be directed towards a deeper understanding of their integrated role, in particular the interaction(s) between these two lifestyle factors from the perspective of sustainable and lifelong health. A future research stream should also investigate how diet type may affect health-related physical fitness and performance parameters, including perceived effort during and after bouts of exercise and recovery time after physical exertion.

## Figures and Tables

**Table 1:** Sample distribution. Values are the number of participants (N) and prevalence (%).

		Total (N)	Male N (%)	Female N (%)	Secondary Level I N (%)	Secondary Level II N (%)
		8799	3249 (36.9)	5550 (63.1)	2651 (30.1)	6148 (69.9)
**Living Environment**						
	Urban	2785	1171 (42.0)	1614 (58.0)	980 (35.2)	1805 (64.8)
	Rural	6014	2078 (34.6)	3936 (65.4)	1671 (27.8)	4343 (72.2)
**Nationality**						
	Austrian	7746	2849 (36.8)	4897 (63.2)	2341 (30.2)	5405 (69.8)
	Other	1053	400 (38.0)	653 (62.0)	310 (29.4)	743 (70.6)
**School Type**						
Middle School		2183	1096 (50.2)	1087 (49.8)	X	
Academic School (AHS, Sec. I)		393	206 (52.4)	187 (47.6)	X	
Prevocational School		236	135 (57.2)	101 (42.8)		X
Vocational School		45	17 (37.8)	28 (62.2)		X
Secondary Technical & Vocational School (BMS, 4 yrs)		1047	414 (39.5)	633 (60.5)		X
Academic School (AHS, Sec. II)		826	278 (33.7)	548 (66.3)		X
Secondary Technical & Vocational School (BHS, 5 yrs)		3777	977 (25.9)	2800 (74.1)		X
Other		292	126 (43.2)	166 (56.8)	75 (25.7)	217 (74.3)

Sec. I—Secondary School level I; Sec. II—Secondary School level I. AHS—Allgemeinbildende Höhere Schule; BMS—Berufsbildende Mittlere Schule (4 years); BHS—Berufsbildende Höhere Schule (5 years). Bold—Total Numbers. X—Type of School grouped in either Secondary School Level I or School Level II.

**Table 2:** Anthropometric characteristics for the total sample and separately for male and female participants as well as secondary level I and secondary level II. Values are means ± SD and prevalence for weight categories.

	Total	Male	Female	Secondary Level I	Secondary Level II
**Age (years) ^1,2,3,4^**	**15.1 ± 2.3**	**14.7 ± 2.3**	**15.4 ± 2.2**	**12.6 ± 1.3**	**16.3 ± 1.6**
Urban	15.0 ± 2.2	14.8 ± 2.2	15.1 ± 2.2	12.7 ± 1.3	16.2 ± 1.6
Rural	15.2 ± 2.3	14.6 ± 2.3	15.5 ± 2.2	12.4 ± 1.3	16.3 ± 1.6
**Body Weight (kg) ^1,2,4,5^**	**58.5 ± 14.3**	**62.4 ± 17.3**	**56.2 ± 11.6**	**49.7 ± 13.1**	**62.2 ± 13.1**
Urban	59.0 ± 15.0	63.7 ± 17.3	55.7 ± 11.9	51.4 ± 14.0	63.2 ± 13.8
Rural	58.2 ± 14.0	61.6 ± 17.2	56.4 ± 11.5	48.7 ± 12.4	61.8 ± 12.8
**Height (cm) ^3,5^**	**166.6 ± 10.5**	**170.6 ± 13.0**	**164.2 ± 7.9**	**159.2 ± 10.7**	**169.8 ± 8.7**
Urban	166.7 ± 10.9	171.2 ± 12.6	163.4 ± 8.1	159.8 ± 10.7	170.4 ± 9.1
Rural	166.5 ± 10.4	170.3 ± 13.2	164.5 ± 7.8	158.8 ± 10.7	169.5 ± 8.6
**BMI_PCT_ ^1,2,3,4,5^**	**49.7 ± 30.4**	**57.3 ± 29.2**	**45.2 ± 30.2**	**52.4 ± 30.8**	**48.5 ± 30.2**
Urban	52.3 ± 30.5	59.4 ± 29.2	47.1 ± 30.5	54.8 ± 30.8	50.9 ± 30.3
Rural	48.5 ± 30.3	56.1 ± 29.2	44.5 ± 30.1	51.0 ± 30.7	47.5 ± 30.1
**Underweight (%) ^1^**	**12.4**	**7.4**	**15.4**	**11.5**	**12.8**
Urban	11.1	6.3	14.6	10.0	11.7
Rural	13.1	8.0	15.8	12.4	13.3
**Normal Weight (%)**	**75.8**	**77.1**	**75.0**	**74.3**	**76.4**
Urban	75.0	76.3	74.1	72.8	76.2
Rural	76.1	77.6	75.3	75.2	76.5
**Overweight (%) ^1,5^**	**7.1**	**9.2**	**5.9**	**8.7**	**6.4**
Urban	8.3	10.3	6.8	9.3	7.7
Rural	6.6	8.6	5.5	8.4	5.9
**Obese (%) ^1,3,4^**	**4.7**	**6.2**	**3.8**	**5.5**	**4.3**
Urban	5.6	7.1	4.6	8.0	4.4
Rural	4.2	5.8	3.4	4.0	4.3

BMI_PCT_—BMI Percentile. ^1^ significant difference between participants living in urban and rural areas across the total sample (*p* < 0.01); ^2^ significant differences between male participants living in urban and rural areas (*p* < 0.01); ^3^ significant differences between female participants living in urban and rural areas (*p* < 0.01); ^4^ significant difference between participants living in urban and rural areas in secondary level I (*p* < 0.01); ^5^ significant difference between participants living in urban and rural areas in secondary level II (*p* < 0.01). Bold—Total Numbers.

**Table 3:** Sports participation by sex, age, living environment, and nationality. Values are the number of participants (N) and prevalence (%) as well as mean with standard deviation for the number of days with sports.

	Leisure-Time Sports N (%)	Club Sports N (%)	Sport Days/Week Mean ± SD
**Total Sample**	**7253 (82.4)**	**3083 (42.5)**	**2.9 ± 2.0**
Male	2787 (85.8)	1448 (52.0)	3.3 ± 2.1
Female	4466 (80.5)	1635 (36.6)	2.6 ± 1.9
**Age Groups**			
10–12 years	1149 (92.7)	592 (51.5)	3.7 ± 2.0
13–14 years	1778 (86.9)	811 (45.6)	3.2 ± 2.0
15–16 years	2435 (79.7)	1022 (42.0)	2.6 ± 2.0
17–19 years	1891 (76.9)	658 (34.8)	2.4 ± 1.9
**Living Environment**			
Urban	2256 (81.0)	1302 (42.3)	2.8 ± 2.0
Rural	4997 (83.1)	2868 (42.6)	2.9 ± 2.0
**Nationality**			
Austria	6456 (83.3)	2820 (43.7)	2.9 ± 2.0
Other	797 (75.7)	263 (33.0)	2.6 ± 2.0

Bold—Total Numbers.

**Table 4:** Anthropometric characteristics by sports participation. Values are presented as means ± SD for the first four variables and prevalence for BMI subgroups.

	Leisure-Time Sports		Club Sports	
	Yes	No	Yes	No
**Age (years) ^1,2^**	15.0 ± 2.3	15.8 ± 2.0	14.7 ± 2.3	15.2 ± 2.3
**Height (cm) ^1^**	166.3 ± 10.8	167.9 ± 9.3	166.5 ± 11.3	166.2 ± 10.4
**Body Weight (kg) ^1,2^**	57.8 ± 14.0	61.5 ± 15.2	56.9 ± 13.8	58.5 ± 14.1
**BMI Percentile**	49.5 ± 30.0	50.6 ± 32.2	48.9 ± 28.8	49.9 ± 30.9
**BMI Subgroups (%)**				
Underweight ^1^	12.0	14.5	11.5	12.4
Normalweight ^1,2^	77.1	69.5	80.1	74.9
Overweight ^1,2^	6.8	8.6	5.4	7.8
Obese ^1,2^	4.1	7.4	3.0	4.9

^1^ significant difference between sports participation during leisure time (*p* < 0.01); ^2^ significant difference between club sports participation (*p* < 0.01).

**Table 5:** Dietary pattern by sex, age, living environment, and nationality. Values are the number of participants (N) and prevalence (%).

	Mixed Diet N (%)	Vegetarian N (%)	Vegan N (%)
**Total Sample**	**7421 (84.3)**	**745 (8.5)**	**633 (7.2)**
Male	2827 (87.0)	148 (4.6)	274 (8.4)
Female	4594 (82.8)	597 (10.7)	359 (6.5)
**Age Groups**			
10–12 years	931 (75.1)	101 (8.2)	207 (16.7)
13–14 years	1723 (84.2)	137 (6.7)	187 (9.1)
15–16 years	2626 (86.0)	276 (9.0)	152 (5.0)
17–19 years	2141 (87.1)	231 (9.4)	87 (3.5)
**Living Environment**			
Urban	2262 (81.2)	244 (8.8)	279 (10.0)
Rural	5159 (85.8)	501 (8.3)	354 (5.9)
**Nationality**			
Austria	6583 (85.0)	660 (8.5)	503 (6.5)
Other	838 (79.6)	85 (8.1)	130 (12.3)

Bold—Total Numbers.

**Table 6:** Anthropometric characteristics by dietary pattern. Values are presented as means ± SD for the first four variables and prevalence for BMI subgroups.

	Mixed Diet	Vegetarian	Vegan
**Age (years) ^2,3^**	15.2 ± 2.2	15.4 ± 2.3	13.8 ± 2.4
**Height (cm) ^1,2,3^**	167.1 ± 10.4	165.6 ± 9.0	162.0 ± 12.4
**Body Weight (kg) ^1,2,3^**	59.1 ± 14.4	56.6 ± 12.2	53.5 ± 14.8
**BMI Percentile ^1,3^**	50.0 ± 30.3	44.5 ± 30.1	51.6 ± 31.7
**BMI Subgroups [n (%)]**			
Underweight ^1,2^	884 (11.9)	116 (15.6)	95 (15.0)
Normal Weight ^2^	5650 (76.1)	561 (75.3)	456 (72.0)
Overweight	525 (7.1)	49 (6.6)	51 (8.1)
Obese ^1,3^	362 (4.9)	19 (2.6)	31 (4.9)

^1^ significant difference between mixed diet and vegetarian diet (*p* < 0.01); ^2^ significant difference between mixed diet and vegan diet (*p* < 0.01); ^3^ significant difference between vegetarian and vegan diet (*p* < 0.01).

**Table 7:** Dietary habits by sports participation. Values are in prevalence (%).

	Leisure-Time Sports		Club Sports		Sport Days/Week		
	Yes	No	Yes	No	None	1–3 Days	4–7 Days
**Daily Fruit ^1,2,3^**	69.9	47.8	72.6	67.9	47.8	65.8	75.2
**Daily vegetable ^1,3^**	66.1	53.5	65.0	67.0	53.5	64.6	75.2
**Fluid Intake (>2 L/day) ^1,2,3^**	24.0	16.8	26.7	22.1	16.8	18.4	31.3
**Most common drink**							
Water ^1,3^	74.3	66.0	73.2	75.2	66.0	73.5	75.5
Diluting Juice ^3^	9.0	9.5	9.7	8.5	9.5	9.9	8.0
Softdrink ^1,3^	3.9	8.3	4.1	3.8	8.3	3.5	4.5
Alcohol ^1,3^	45.6	53.0	44.5	46.3	53.0	49.3	40.7
Smoking ^1,2,3^	7.6	17.3	6.6	8.2	17.3	7.8	7.2

^1^ significant difference between sports participation during leisure time (*p* < 0.01); ^2^ significant difference between club sports participation (*p* < 0.01); ^3^ significant difference between sport days/week (*p* < 0.01).

**Table 8:** Health behaviors by dietary pattern. Values are in prevalence (%).

	Mixed Diet	Vegetarian	Vegan
**Leisure-time sports participation ^2^**	82.0	83.6	86.4
**Club sports participation**	42.5	41.9	43.5
**Fluid Intake (>2 L/day)**	23.0	18.9	24.2
**Most common drink**			
Water ^1,2,3^	72.8	80.8	64.1
Diluting juice ^1,2^	9.9	3.8	6.0
Softdrink ^1,3^	4.8	2.7	6.5
Alcohol ^2,3^	48.8	46.3	25.3
Smoking	9.1	9.0	10.9

^1^ significant difference between mixed diet and vegetarian diet (*p* < 0.01); ^2^ significant difference between mixed diet and vegan diet (*p* < 0.01); ^3^ significant difference between vegetarian and vegan diet (*p* < 0.01).

## Data Availability

The data are not publicly available due to data protection and security laws.

## Appendix A

Results supplemental to the main text are provided within four Table A1, Table A2, Table A3 and Table A4.

**Table A1:** Anthropometric Characteristics by federal state and living area in secondary level I pupils. Values are means ± SD and prevalence for weight categories.

	N	Age (Years)	Height (cm)	Body Weight (kg)	BMI_PCT_ (kg/m^2^)	Overweight/ Obesity (%)	Underweight (%)
**Burgenland**	**286**	**12.4 ± 1.2**	**159.4 ± 10.1**	**50.4 ± 12.5**	**56.4 ± 30.7**	**17.5**	**9.8**
Urban	70	12.5 ±1.2	160.7 ± 8.3	53.4 ± 12.2	62.3 ± 28.3	22.9	4.3
Rural	216	12.4 ± 1.2	158.9 ± 10.5	49.5 ± 12.4	54.4 ± 31.3	15.7	11.6
**Carinthia**	**269**	**12.5 ± 1.2**	**158.8 ± 11.1**	**49.3 ± 13.1**	**51.6 ± 31.9**	**14.5**	**14.5**
Urban	139	12.5 ± 1.3	159.7 ± 10.6	50.2 ± 13.7	52.2 ± 31.9	16.5	13.7
Rural	130	12.5 ± 1.1	157.8 ± 11.7	48.2 ± 12.4	51.0 ± 32.1	12.3	15.4
**Lower Austria**	**532**	**12.6 ± 1.4**	**158.8 ± 11.2**	**50.4 ± 13.9**	**55.0 ± 31.3**	**16.2**	**9.6**
Urban	146	12.9 ± 1.5	158.6 ± 11.3	51.7 ± 14.8	56.4 ± 31.3	17.1	8.2
Rural	386	12.5 ± 1.3	158.8 ± 11.2	49.9 ± 13.5	54.5 ± 31.3	15.8	10.1
**Salzburg**	**23**	**13.3 ± 1.3**	**168.0 ± 15.9**	**55.0 ± 12.9**	**52.3 ± 31.6**	**13.0**	**17.4**
Urban	10	13.7 ± 0.8	166.1 ± 13.7	55.3 ± 15.8	50.7 ± 31.3	20.0	20.0
Rural	13	13.0 ± 1.6	169.5 ± 17.9	54.8 ± 10.9	53.6 ± 33.1	7.7	15.4
**Styria**	**284**	**12.6 ± 1.1**	**160.3 ± 10.2**	**50.2 ± 12.6**	**51.9 ± 29.8**	**12.3**	**10.6**
Urban	35	12.3 ± 1.5	156.3 ± 12.1	48.2 ± 13.4	54.5 ± 30.5	17.1	8.6
Rural	249	12.7 ± 1.0	160.9 ± 9.8	50.5 ± 12.5	51.5 ± 29.8	11.6	10.8
**Tyrol**	**229**	**12.6 ± 1.4**	**158.7 ± 11.2**	**48.6 ± 13.7**	**49.2 ± 30.0**	**10.5**	**13.1**
Urban	84	12.8 ± 1.5	158.8 ± 12.1	49.4 ± 15.3	48.3 ± 32.1	11.9	17.9
Rural	145	12.5 ± 1.3	158.7 ± 10.6	48.1 ± 12.7	49.8 ± 28.8	9.7	10.3
**Upper Austria**	**473**	**12.4 ± 1.3**	**158.5 ± 9.9**	**47.7 ± 11.6**	**48.6 ± 30.2**	**11.2**	**14.0**
Urban	69	13.2 ± 0.8	163.7 ± 7.7	53.7 ± 12.2	53.7 ± 30.7	17.4	13.0
Rural	404	12.3 ± 1.3	157.6 ± 10.0	46.6 ± 11.2	47.7 ± 30.1	10.1	14.1
**Vienna**	**180**	**12.5 ± 1.3**	**159.5 ± 11.4**	**48.8 ± 14.8**	**47.6 ± 30.2**	**11.1**	**13.3**
Urban	169	12.5 ± 1.2	159.3 ± 11.6	48.9 ± 15.1	48.5 ± 30.1	11.8	11.8
Rural	11	13.0 ± 1.0	162.0 ± 6.8	46.3 ± 8.5	33.7 ± 28.3	N/A	36.4
**Vorarlberg**	**375**	**12.8 ± 1.4**	**159.7 ± 10.1**	**51.4 ± 13.1**	**55.6 ± 30.5**	**17.6**	**9.1**
Urban	258	12.9 ± 1.4	160.2 ± 9.8	53.3 ± 13.3	60.0 ± 29.6	21.3	5.8
Rural	117	12.5 ± 1.3	158.4 ± 10.5	47.1 ± 11.6	45.9 ± 30.5	9.4	16.2

Bold—Total Numbers.

**Table A2:** Anthropometric Characteristics by federal state and living area in secondary level II pupils. Values are means ± SD and prevalence for weight categories.

	N	Age (Years)	Height (cm)	Body Weight (kg)	BMI_PCT_ (kg/m^2^)	Overweight/ Obesity (%)	Underweight (%)
**Burgenland**	**1037**	**16.2 ± 1.5**	**170.5 ± 9.1**	**63.3 ± 13.9**	**49.5 ± 30.8**	**12.7**	**13.1**
Urban	170	16.0 ± 1.5	170.7 ± 9.4	62.6 ± 14.2	48.5 ± 30.3	12.9	12.9
Rural	867	16.3 ± 1.5	170.5 ± 9.0	63.4 ± 13.9	49.7 ± 30.8	12.7	13.1
**Carinthia**	**137**	**15.4 ± 1.6**	**170.7 ± 10.0**	**60.5 ± 13.0**	**47.7 ± 29.5**	**5.8**	**15.3**
Urban	27	15.1 ± 1.2	166.1 ± 12.0	53.3 ± 9.8	36.5 ± 29.8	N/A	25.9
Rural	110	15.5 ± 1.7	171.8 ± 9.1	62.2 ± 13.1	50.5 ± 29.0	7.3	12.7
**Lower Austria**	**736**	**16.4 ± 1.6**	**170.5 ± 9.0**	**64.3 ± 13.4**	**52.7 ± 30.6**	**13.6**	**10.3**
Urban	143	16.4 ± 1.6	170.7 ± 9.2	63.2 ± 12.1	50.7 ± 29.4	10.5	10.5
Rural	593	16.4 ± 1.6	170.5 ± 9.0	64.6 ± 13.7	53.1 ± 30.9	14.3	10.3
**Salzburg**	**684**	**16.2 ± 1.6**	**168.6 ± 7.9**	**60.6 ± 12.2**	**45.6 ± 30.0**	**9.4**	**13.9**
Urban	107	16.8 ± 1.8	168.6 ± 7.8	60.1 ± 11.3	41.3 ± 30.6	8.4	19.6
Rural	577	16.0 ± 1.5	168.7 ± 7.9	60.7 ± 12.4	46.4 ± 29.4	9.5	12.8
**Styria**	**611**	**16.1 ± 1.6**	**169.0 ± 8.3**	**61.6 ± 12.4**	**49.1 ± 30.6**	**11.3**	**13.7**
Urban	180	15.8 ± 1.4	169.4 ± 8.3	61.2 ± 12.6	49.3 ± 29.8	10.0	11.7
Rural	431	16.2 ± 1.6	168.9 ± 8.3	61.7 ± 12.3	49.0 ± 31.0	11.8	14.6
**Tyrol**	**1041**	**16.3 ± 1.6**	**168.6 ± 8.0**	**60.0 ± 11.2**	**44.0 ± 28.7**	**7.7**	**14.7**
Urban	186	16.5 ± 1.6	168.7 ± 8.1	71.8 ± 11.9	48.4 ± 30.7	12.9	14.0
Rural	855	16.3 ± 1.6	168.5 ± 7.9	59.6 ± 11.0	43.0 ± 28.2	6.5	14.9
**Upper Austria**	**836**	**16.5 ± 1.5**	**168.5 ± 8.3**	**61.3 ± 12.3**	**46.2 ± 29.6**	**9.3**	**12.1**
Urban	233	16.4 ± 1.5	167.8 ± 7.6	61.6 ± 12.1	49.6 ± 30.1	12.0	11.2
Rural	603	16.6 ± 1.5	168.8 ± 8.5	61.2 ± 12.3	44.9 ± 29.3	8.3	12.4
**Vienna**	**648**	**16.1 ± 1.6**	**172.5 ± 9.5**	**65.9 ± 14.9**	**55.7 ± 30.0**	**14.4**	**9.7**
Urban	623	16.1 ± 1.6	172.6 ± 9.5	66.2 ± 15.1	56.1 ± 30.1	14.8	9.6
Rural	25	15.7 ± 1.4	169.2 ± 8.3	59.1 ± 8.4	45.1 ± 26.7	4.0	12.0
**Vorarlberg**	**418**	**16.3 ± 1.6**	**170.2 ± 9.3**	**62.0 ± 13.9**	**47.9 ± 30.1**	**8.9**	**14.4**
Urban	136	16.4 ± 1.7	169.7 ± 9.7	62.1 ± 15.2	48.8 ± 28.6	7.4	9.6
Rural	282	16.2 ± 1.6	170.4 ± 9.1	61.9 ± 13.2	47.4 ± 30.8	9.6	16.7

Bold—Total Numbers.

**Table A3:** Health behavior by federal state and living area in secondary level I students. Values are in prevalences (%) as well as means ± SD for days with sports.

	N	Leisure Time Sports (%)	Club-Sports (%)	Days/Week with Sport (mean ± SD)	Daily Fruits (%)	Daily Veggies (%)	Fluid Intake > 2 L/Day (%)	Water Most Common Fluid (%)	Vegetarian/ Vegan (%)	Alcohol (%)	Smoking (%)
**Burgenland**	**286**	**86.7**	**49.6**	**3.4 ± 2.1**	**75.5**	**59.8**	**21.7**	**71.0**	**24.1**	**13.3**	**8.0**
Urban	70	81.4	47.4	3.1 ±2.1	68.6	64.3	20.0	70.0	24.3	15.7	10.0
Rural	216	88.4	50.3	3.6 ± 2.1	77.8	58.3	22.2	71.3	24.1	12.5	7.4
**Carinthia**	**269**	**89.2**	**37.5**	**3.6 ± 2.1**	**78.4**	**56.1**	**20.8**	**67.3**	**28.3**	**6.3**	**4.1**
Urban	139	90.6	39.7	3.4 ± 1.9	79.1	58.8	18.7	66.9	30.2	7.9	5.8
Rural	130	87.7	35.1	3.8 ± 2.3	77.7	53.8	23.1	67.7	26.2	4.6	2.3
**Lower Austria**	**532**	**91.4**	**38.9**	**3.6 ± 2.0**	**73.9**	**59.2**	**23.7**	**63.5**	**23.5**	**8.6**	**3.4**
Urban	146	86.3	36.5	3.3 ± 2.1	69.2	54.8	24.7	59.6	32.9	10.3	5.5
Rural	386	93.3	39.7	3.7 ± 1.9	75.6	60.9	23.3	65.0	19.9	8.0	2.6
**Salzburg**	**23**	**95.7**	**59.1**	**3.3 ± 1.8**	**56.5**	**34.8**	**30.4**	**69.6**	**17.4**	**26.1**	**4.3**
Urban	10	90.0	88.9	2.8 ± 1.9	70.0	30.0	20.0	70.0	20.0	20.0	0.0
Rural	13	100.0	38.5	3.8 ± 1.7	46.2	38.5	38,5	69.2	15.4	30.8	7.7
**Styria**	**284**	**92.3**	**49.6**	**3.6 ± 2.0**	**75.0**	**59.9**	**18.7**	**61.6**	**18.3**	**7.0**	**3.5**
Urban	35	94.3	54.5	3.6 ± 1.7	88.6	68.6	20.0	80.0	40.0	2.9	0.0
Rural	249	92.0	48.9	3.6 ± 2.1	73.1	58.6	18.5	59.0	15.3	7.6	4.0
**Tyrol**	**229**	**93.0**	**54.9**	**3.7 ± 1.9**	**71.6**	**62.0**	**23.1**	**63.8**	**19.7**	**7.9**	**2.2**
Urban	84	88.1	52.7	3.2 ± 1.9	71.4	71.4	27.4	65.5	28.6	8.3	3.6
Rural	145	95.9	56.1	3.9 ± 1.8	71.7	56.6	20.7	62.8	14.5	7.6	1.4
**Upper Austria**	**473**	**93.4**	**53.4**	**3.7 ± 2.0**	**79.1**	**63.4**	**26.6**	**66.4**	**17.5**	**6.3**	**1.5**
Urban	69	87.0	51.7	3.2 ± 2.1	75.3	58.0	27.5	65.2	17.4	5.8	1.4
Rural	404	94.6	53.7	3.8 ± 2.0	79.7	64.4	26.5	66.6	17.6	6.4	1.5
**Vienna**	**180**	**91.1**	**47.6**	**3.4 ± 2.0**	**72.2**	**73.3**	**20.6**	**85.6**	**15.0**	**4.4**	**1.1**
Urban	169	92.9	47.8	3.4 ± 1.9	72.2	72.8	21.3	85.8	16.0	4.1	0.6
Rural	11	63.6	42.9	2.9 ± 2.9	72.7	81.8	9.1	81.8	N/A	9.1	9.1
**Vorarlberg**	**375**	**83.5**	**62.3**	**3.0 ± 2.1**	**73.1**	**63.7**	**22.9**	**72.0**	**25.6**	**10.9**	**4.5**
Urban	258	81.0	55.5	2.9 ± 2.1	70.9	64.3	20.5	70.9	29.1	12.0	6.2
Rural	117	88.9	76.0	3.3 ± 2.0	77.8	62.4	28.2	74.4	17.9	8.5	0.9

Bold—Total Numbers.

**Table A4:** Health behavior by federal state and living area in secondary level II students. Values are in prevalence (%) as well as means ± SD for days with sports.

	N	Leisure Time Sports (%)	Club-Sports (%)	Days/Week with Sport (Mean ± SD)	Daily Fruits (%)	Daily Veggies (%)	Fluid Intake >2 L/day (%)	Water Most Common Fluid (%)	Vegetarian/ Vegan (%)	Alcohol (%)	Smoking (%)
**Burgenland**	**1037**	**74.5**	**42.8**	**2.5 ± 2.0**	**60.7**	**60.8**	**24.2**	**77.4**	**13.4**	**71.2**	**15.7**
Urban	170	76.5	43.8	2.6 ± 2.0	65.9	58.8	27.6	77.6	16.5	65.3	14.1
Rural	867	74.2	42.6	2.5 ± 2.0	59.6	61.1	23.5	77.4	12.8	72.3	16.0
**Carinthia**	**137**	**81.0**	**33.3**	**3.0 ± 2.0**	**62.0**	**56.2**	**27.0**	**65.0**	**12.4**	**51.1**	**14.6**
Urban	27	77.8	47.6	3.1 ± 2.1	66.7	74.1	18.5	59.3	18.5	48.1	7.4
Rural	110	81.8	30.0	2.9 ± 2.0	60.9	51.8	29.1	66.4	10.9	51.8	16.4
**Lower Austria**	**736**	**73.9**	**36.8**	**2.4 ± 2.0**	**55.3**	**61.4**	**25.4**	**73.8**	**11.4**	**68.1**	**14.5**
Urban	143	72.0	37.9	2.7 ± 2.2	58.0	62.9	23.1	77.6	14.7	55.2	16.8
Rural	593	74.4	36.6	2.3 ± 1.9	54.6	61.0	26.0	72.8	10.6	71.2	14.0
**Salzburg**	**684**	**80.8**	**35.4**	**2.4 ± 1.8**	**63.9**	**63.5**	**21.8**	**77.0**	**12.1**	**66.2**	**12.4**
Urban	107	80.4	38.4	2.4 ± 1.9	58.9	68.2	22.4	78.5	17.8	50.3	16.8
Rural	577	80.9	34.9	2.4 ± 1.8	64.8	62.6	21.7	76.8	11.1	68.8	11.6
**Styria**	**611**	**79.7**	**41.9**	**2.7 ± 2.0**	**62.0**	**64.5**	**22.7**	**69.4**	**15.5**	**60.7**	**10.5**
Urban	180	76.1	40.9	2.7 ± 2.1	67.2	63.3	26.1	76.1	18.9	45.0	13.9
Rural	431	81.2	42.3	2.7 ± 2.0	59.9	65.0	21.3	66.6	14.2	67.3	9.0
**Tyrol**	**1041**	**86.6**	**40.8**	**2.9 ± 1.9**	**70.5**	**70.5**	**19.1**	**76.0**	**11.6**	**66.4**	**9.2**
Urban	186	88.7	36.4	2.8 ± 1.7	68.3	71.5	18.8	74.7	11.8	50.5	12.9
Rural	855	86.2	41.8	3.0 ± 1.9	71.0	70.3	19.2	76.3	11.6	69.8	8.4
**Upper Austria**	**836**	**79.2**	**34.7**	**2.3 ± 1.8**	**63.2**	**70.3**	**20.9**	**73.8**	**14.2**	**69.3**	**9.8**
Urban	233	76.0	32.8	2.4 ± 1.9	55.4	64.8	19.3	71.2	17.6	50.2	11.6
Rural	603	80.4	35.5	2.2 ± 1.8	66.2	72.5	21.6	74.8	12.9	76.6	9.1
**Vienna**	**648**	**77.2**	**35.6**	**2.5 ± 1.9**	**55.9**	**62.5**	**23.9**	**75.0**	**11.7**	**36.6**	**10.6**
Urban	623	77.0	35.8	2.4 ± 1.9	56.5	62.1	23.9	75.3	11.4	37.1	10.6
Rural	25	80.0	30.0	3.0 ± 2.4	40.0	72.0	24.0	68.0	20.0	24.0	12.0
**Vorarlberg**	**418**	**79.2**	**50.8**	**2.8 ± 2.0**	**62.2**	**67.5**	**25.1**	**80.4**	**16.0**	**62.0**	**8.4**
Urban	136	77.9	55.7	2.7 ± 2.0	57.4	69.1	27.9	77.2	15.4	61.0	8.8
Rural	282	79.8	48.4	2.8 ± 2.1	64.5	66.7	23.8	81.9	16.3	62.4	8.2

Bold—Total Numbers.
